# First Pass Success Without Adverse Events Is Reduced Equally with Anatomically Difficult Airways and Physiologically Difficult Airways

**DOI:** 10.5811/westjem.2020.10.48887

**Published:** 2021-02-01

**Authors:** Garrett S. Pacheco, Nicholas B. Hurst, Asad E. Patanwala, Cameron Hypes, Jarrod M. Mosier, John C. Sakles

**Affiliations:** * University of Arizona College of Medicine, Department of Emergency Medicine, Tucson, Arizona; † The University of Sydney School of Pharmacy, Royal Prince Alfred Hospital, NSW, Sydney, Australia; ‡ University of Arizona College of Medicine, Department of Medicine, Division of Pulmonary, Allergy, Critical Care and Sleep, Tucson, Arizona

## Abstract

**Introduction:**

The goal of emergency airway management is first pass success without adverse events (FPS-AE). Anatomically difficult airways are well appreciated to be an obstacle to this goal. However, little is known about the effect of the physiologically difficult airway with regard to FPS-AE. This study evaluates the effects of both anatomically and physiologically difficult airways on FPS-AE in patients undergoing rapid sequence intubation (RSI) in the emergency department (ED).

**Methods:**

We analyzed prospectively recorded intubations in a continuous quality improvement database between July 1, 2014–June 30, 2018. Emergency medicine (EM) or emergency medicine/pediatric (EM-PEDS) residents recorded patient, operator, and procedural characteristics on all consecutive adult RSIs performed using a direct or video laryngoscope. The presence of specific anatomically and physiologically difficult airway characteristics were also documented by the operator. Patients were analyzed in four cohorts: 1) no anatomically or physiologically difficult airway characteristics; 2) one or more anatomically difficult airway characteristics; 3) one or more physiologically difficult airway characteristics; and 4) both anatomically and physiologically difficult airway characteristics. The primary outcome was FPS-AE. We performed a multivariable logistic regression analysis to determine the association between anatomically difficult airways or physiologically difficult airways and FPS-AE.

**Results:**

A total of 1513 intubations met inclusion criteria and were analyzed. FPS-AE for patients without any difficult airway characteristics was 92.4%, but reduced to 82.1% (difference = −10.3%, 95% confidence interval (CI), −14.8% to −5.6%) with the presence of one or more anatomically difficult airway characteristics, and 81.7% (difference = −10.7%, 95% CI, −17.3% to −4.0%) with the presence of one or more physiologically difficult airway characteristics. FPS-AE was further reduced to 70.9% (difference = −21.4%, 95% CI, −27.0% to −16.0%) with the presence of both anatomically and physiologically difficult airway characteristics. The adjusted odds ratio (aOR) of FPS-AE was 0.37 [95% CI, 0.21 – 0.66] in patients with anatomically difficult airway characteristics and 0.36 [95% CI, 0.19 – 0.67] for patients with physiologically difficult airway characteristics, compared to patients with no difficult airway characteristics. Patients who had both anatomically and physiologically difficult airway characteristics had a further decreased aOR of FPS-AE of 0.19 [95% CI, 0.11 – 0.33].

**Conclusion:**

FPS-AE is reduced to a similar degree in patients with anatomically and physiologically difficult airways. Operators should assess and plan for potential physiologic difficulty as is routinely done for anatomically difficulty airways. Optimization strategies to improve FPS-AE for patients with physiologically difficult airways should be studied in randomized controlled trials.

## INTRODUCTION

First pass success during tracheal intubation is associated with fewer adverse events in patients with critical illness.^1^,^2^ Anatomic characteristics that impede glottic visualization or tube passage increase the risk of adverse events.^1^,^3^ Consequently, emergency airway management has primarily focused on the prediction and management of patients with anatomically difficult airways to optimize the odds of achieving first pass success without adverse events (FPS-AE). Recently, there has been an increasing awareness of the importance of the physiologically difficult airway, which may increase the risk of AEs independent of any anatomic difficulty.^4^–^7^ Critically ill patients are at high risk of hypoxemia, hypotension, and cardiac arrest because of deranged physiology that is often exacerbated during or resulting from airway management.^5^,^8^ Hypoxemia and hypotension are particularly hazardous risks for cardiac arrest.^9^–^13^

Unfortunately, data are lacking regarding the effect of physiologically difficult airways on FPS-AE in the emergency department (ED). This study explores the association of physiologically and anatomically difficult airways with FPS-AE. It was hypothesized that physiologically difficult airways would also reduce FPS-AE.

## METHODS

### Study Design

We prospectively collected data on patient and operator characteristics for every intubation performed in our ED and stored the data in a continuous quality improvement (CQI) database, which has been described previously.^1^,^14^ In 2014 we updated our airway data collection form to include characteristics related to the physiologically difficult airway. This is a retrospective analysis of that CQI data between July 1, 2014–June 30, 2018. This project was granted exemption from informed consent requirements by The University of Arizona Institutional Review Board.

### Study Setting and Population

This study was conducted at a 61-bed, tertiary care, urban ED with an annual volume of approximately 80,000 visits. The ED is a Level I trauma center designated by the American College of Surgeons and supports two separate three-year emergency medicine (EM) residency programs and a five-year combined EM/Pediatrics (EM-PEDS) residency program. Nearly all intubations are performed by EM or EM-PEDS residents under the direct supervision of an EM or EM-PEDS attending physician who is ultimately responsible for all aspects of emergency airway management. Inclusion criteria included all patients intubated by rapid sequence intubation (RSI). We excluded pediatric patients (age <18 years), and patients not intubated with either direct laryngoscopy (DL) or video laryngoscopy (VL). Pediatric patients were excluded because they have age-specific vital signs and compensatory mechanisms limiting the comparison to adults. Intubations performed by non-EM providers were not included because of their varying degrees of airway training. EM and EM-PEDS residents all receive annual training through similar airway didactics and rigorous simulation laboratory experience. Methods of intubation other than RSI were excluded from this analysis to create a homogenous cohort of patients.

**Population Health Research Capsule**

What do we already know about this issue?

The physiologically difficult airway draws attention to non-anatomic difficulties that can increase the risks associated with emergency airway management.

What was the research question?

We sought to explore the relationship between physiologically and anatomically difficult airways and the desired outcome of first pass success without adverse events (FPS-AE).

What was the major finding of the study?

The physiologically difficult airway was associated with a similar reduction in FPS-AE compared to the anatomically difficult airway.

How does this improve population health?

Recognizing the physiologically difficult airway will encourage clinicians to identify and optimize physiologic derangements before intubation.

### Data Acquisition

After each intubation, operators complete a data collection form that includes indication for, method of, and devices used for intubation, Cormack-Lehane view obtained, number and outcome of each attempt, and adverse events. An intubation attempt was defined as insertion of the laryngoscope into the mouth, regardless of whether an attempt was made to insert a tracheal tube. Adverse events include oxygen desaturation, hypotension, esophageal or mainstem intubation, aspiration, unintentional extubation, cuff damage, pneumothorax, dental/airway trauma, dysrhythmia, laryngospasm, and cardiac arrest.^1^ Hypoxemia and hypotension were not considered adverse events if these physiologic derangements were present before airway management commenced. They were only considered adverse events if these physiologic derangements improved prior to the intubation attempt and subsequently deteriorated. Anatomically and physiologically difficult airway characteristics are listed in Table 1. As described previously, missing data forms are identified through a structured cross-referencing workflow to ensure 100% capture.^1^

### Outcomes and Definitions

The primary outcome was the proportion of patients with FPS-AE, which we defined as successful tracheal intubation on a single laryngoscope insertion without an AE. The secondary outcome was the incidence of AEs for each cohort.

### Data Analysis

Patients were categorized into four groups: 1) patients with no difficult airway characteristics; 2) patients with one or more anatomically difficult airway characteristics; 3) patients with one or more physiologically difficult airway characteristics; and 4) patients with both anatomically and physiologically difficult airway characteristics. We reported patient demographic and clinical characteristics descriptively using means (SD) or medians (IQR), as appropriate for continuous variables. Categorical variables were reported as frequencies with percentages. The primary outcome of FPS-AE was compared between groups using a Fisher’s exact test. We conducted a multivariable logistic regression analysis to determine the association between type of difficult airway characteristics and the outcome of FPS-AE. Potential confounding variables included in the model were defined a priori based on previous literature and clinical expertise of the investigators.^1^ These included operator postgraduate year level, trauma status, age, and device used. The model was checked for interactions. Linearity in the log-odds was checked for continuous variables. We assessed model fit using the Hosmer-Lemeshow goodness of fit test. All statistical analyses were performed with STATA version 15 (StataCorp, College Station, TX).

## RESULTS

**Figure 1:**
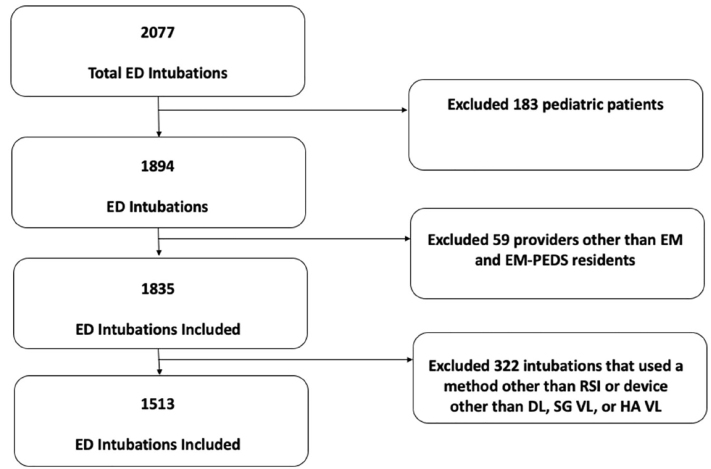
Flowchart of patients in the study during the four-year period. *ED*, emergency department; *EM*, emergency medicine residents; *EM-PEDS*, combined emergency medicine and pediatric residents; *RSI*, rapid sequence intubation; *DL*, direct laryngoscopy; *SG VL*, standard geometry video laryngoscope; *HA VL*, hyperangulated video laryngoscope.

A total of 2,077 intubations were performed during the study period, of which 1,513 patients met the inclusion criteria and were included in the analysis (Figure 1). Demographics are presented in Table 2. Of patients with an anatomically difficult airway, 49% (321/649) were intubated for a traumatic injury and more commonly by senior residents (44%, 284/649), while only 3% (5/186) of patients with a physiologically difficult airway were intubated for a traumatic injury and by a senior resident in 38% (70/186) of encounters. Only 5.3% of patients with anatomically difficult airways received ketamine for induction compared to 20.5% of physiologically difficult airway patients who received ketamine for induction. Of patients with an anatomically difficult airway 44% (288/649) were intubated with a hyperangulated video laryngoscope (HA VL. whereas only 12% (23/186) of patients with a physiologically difficult airway were intubated with a HA VL.

FPS-AE was reduced by similar amounts in both anatomically and physiologically difficult airway groups. This was additive when both difficult airway characteristics were present (Please see Table 3). The adjusted odds ratio (aOR) of FPS-AE are presented in Table 4. The model fit the data well (Hosmer-Lemeshow goodness of fit, *p* = 0.302), and no interactions were identified. The aOR of FPS-AE for individual difficult airway characteristics are presented in Table 5. Anatomically difficult airway characteristics that were negatively associated with FPS-AE were blood in the airway, small mandible, large tongue, and restricted mouth opening. Hypoxemia was the only physiologically difficult airway characteristic found to be negatively associated with FPS-AE.

**Figure 2:**
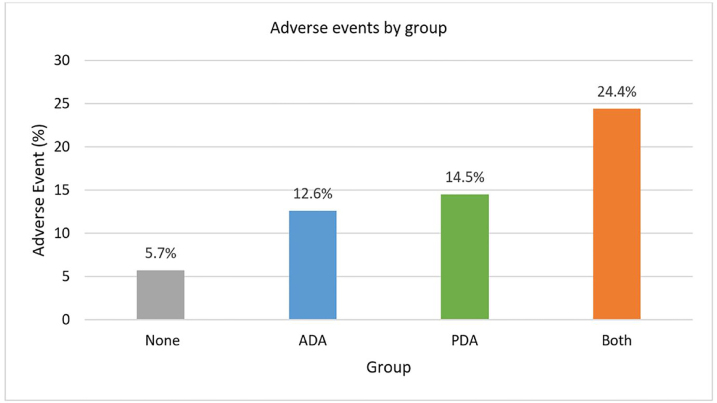
Adverse events by group. *None*, no difficult airway characteristic; *ADA*, anatomically difficulty airway; *PDA*, physiologically difficult airway; *Both*, presence of both anatomically and physiologically difficult airway characteristics.

The incidence of adverse events by cohort is depicted in Figure 2. The prevalence of AEs was similar between the anatomically and physiologically difficult airway cohorts. Oxygen desaturation was the most frequent AE in patients with anatomically difficult airways occurring in 9.2% (60/649) of tracheal intubations. In this group, hypotension occurred in only 1% (6/649) of patients. Oxygen desaturation (8.6%; 16/186) was also the most common AE in the physiologically difficult airway cohort. In addition, hypotension accounted for a significant number of AEs (6.4%; 12/186).

## DISCUSSION

Patients undergoing emergency airway management in the ED are at considerable risk of failed intubation attempts and AEs, which can negatively impact patient care. Thus, the objective of ED intubation is to accomplish FPS-AE to maximize patient safety. It is well appreciated that anatomically difficult airways can hinder attempts to achieve this objective. However, little is known about the impact of physiologically difficult airways on this objective. The aim of this investigation was to ascertain and compare the impact of anatomically and physiologically difficult airways on FPS-AE. We found that anatomically and physiologically difficult airways decreased FPS-AE virtually to the same extent. With the presence of one or more anatomically difficult airway characteristics, FPS-AE was reduced to 82.1%, while the presence of one or more physiologically difficult airway characteristics reduced FPS-AE to 81.7%.

Additional analysis demonstrated specific characteristics of difficult airways that were associated with reduction of FPS-AE. The logistic regression analysis revealed a reduced aOR for the following anatomically difficult airway characteristics: blood in the airway (0.52 [95% CI, 0.35 – 0.78]); small mandible (0.56 [95% CI, 0.35 – 0.89]); large tongue (0.44 [95% CI, 0.29 – 0.65]); and restricted mouth opening (0.32 [95% CI, 0.21 – 0.50]). Interestingly, the only physiologically difficult airway characteristic in the logistic regression analysis found to be associated with a reduction of FPS-AE was hypoxemia (0.35 [95% CI, 0.26 – 0.48]). A possible explanation for this is that hypoxemia is the one physiologically difficult airway characteristic that can impact both variables in the FPS-AE outcome.

For example, patients with pre-existent hypoxemia due to intrapulmonary shunt are at great risk for rapid oxygen desaturation with intubation. Thus, if the oxygen saturation decreases below 90% this would immediately result in an AE. If the oxygen saturation were above 90% but rapidly decreasing, the airway operator might choose to abort that attempt to reoxygenate, thus impacting the first pass success variable of the outcome. Also, since oxygen saturation is a continuously monitored variable throughout intubation, it more than any other physiologically difficult airway characteristic is more likely to influence the operator to abort laryngoscopy. It is possible that due to the low prevalence of the other physiologically difficult airway characteristics we were unable to demonstrate a statistically significant reduction in FPS-AE. Larger studies would likely be needed. The physiologically difficult airway, particularly hypoxemia, can negatively impact patient outcomes following emergency airway management.

While the purpose of this study was to investigate the anatomically and physiologically difficult airways impact on FPS-AE, an additional variable was found to influence the results. Both standard geometry video laryngoscopes (SG VL, aOR 1.77 [95% CI, 1.04 – 3.03]) and HA VL (aOR 1.74 [95% CI, 1.00 – 3.02]) were associated with an increase in FPS-AE. However, additional variables such as airway operator experience, the presence of traumatic injury, or age of the patient had no effect on FPS-AE. As seen in Table 2, this ED primarily uses VL to intubate patients. Our institution has been using VL in the ED for the last 20 years. The airway operators are very comfortable with the devices and are skilled with them. They are often used as the routine airway device. Numerous studies from this ED have demonstrated improved first-pass success while using VL.^1^,^15^–^18^ Considering the variables assessed that may directly impact FPS-AE, VL was the only variable significant that may positively effect FPS-AE.

The data from the current study show an association between physiologically difficult airways and decreased FPS-AE. As stated above, the decrease in FPS-AE was of the same magnitude for patients with anatomically difficult airways as those with physiologically difficult airways. Focused interventions directed at attenuating these physiologically difficult airway characteristics have potential to improve the safety of emergency airway management. Implementation of an intubation bundle for the intensive care unit (ICU) was associated with a 50% reduction in the incidence of severe hypoxemia and cardiovascular collapse.^19^ This bundle emphasized attenuating these physiological risks, including using noninvasive positive-pressure ventilation (NIPPV) for preoxygenation, which reduced the risk of hypoxemia.

Positive pressure ventilation for preoxygenation in hypoxemic patients (oxygen saturation <93%) was recently implemented in an air medical service, which resulted in reduced hypoxemia, increased intubation success, and had no effect on the incidence of witnessed aspiration.^20^ Another prehospital study evaluated the impact of aggressive preoxygenation coupled with apneic oxygenation on hypoxemic events during emergency airway management. Their intubation bundle included upright positioning, positive pressure ventilation in hypoxemic patients, delayed sequence intubation, and apneic oxygenation. This combination was associated with a reduction of hypoxemic episodes from 44.2% to 3.5%.^21^ Baillard demonstrated that the use of NIPPV for intubations in the ICU reduced desaturation rates (7% vs 46%) compared to standard face mask preoxygenation.^22^ These studies demonstrate the importance of advanced preoxygenation techniques in these patients with physiologically difficult airways to attenuate the risks.

In addition to the use of positive pressure for preoxygenation, high-flow nasal oxygen (HFNO) has been demonstrated as a technique to further enhance preoxygenation.^23^ However, in patients with severe hypoxemia, NIPPV prevented desaturation more frequently compared to HFNO in patients who underwent RSI.^24^ The addition of HFNO to NIPPV further decreased the incidence of oxygen desaturation to 4% compared to 21% of patients preoxygenated with non-invasive ventilation alone during tracheal intubation of critically ill patients.^25^

Hypoxemia and hypotension are the two most frequent physiologic disturbances that contribute to serious AEs during airway management of the critically ill. Although there are several proposed approaches to prepare and proceed with intubation in the severely hypoxemic physiologically difficult airway, evidence for interventions to reduce peri-intubation hypotension is limited. Administration of norepinephrine immediately post-intubation (in hypotensive patients) and the avoidance of potent sympatholytic induction agents that depress cardiovascular function (eg, propofol and thiopental) reduced the number of cardiac arrest episodes.^19^ The same patient group also received fluid loading as part of their 10-point intubation bundle. However, a recent trial with a heterogenous group of patients in eight ICUs and one ED who received a 500-mL fluid bolus before induction did not demonstrate any significant reduction in hemodynamic complications.^26^ It is possible that fluid administration may have no effect on patients who have already had an initial fluid resuscitation prior to their ICU intubation. Generally, fluid resuscitation remains the recommendation as clinically indicated to the volume-responsive hypovolemic patient and, if necessary, administering inopressor agents prior to intubation for hemodynamic optimization of the physiologically difficult airway patient.

The medication used for induction may also contribute to peri-intubation hypotension. As mentioned, sympatholytic agents that contribute to depressed cardiovascular function including benzodiazepines, propofol, and thiopental should be avoided in patients with physiologically difficult airways.^5^,^19^ Ketamine has sympathomimetic properties and etomidate is considered hemodynamically neutral, making these agents attractive choices for patients with hemodynamic compromise prior to intubation. Our study revealed a preference for these agents as 78% of patients with physiologically difficult airways received etomidate for induction and 20.5% received ketamine for induction. Ketamine use has demonstrated mixed results with some studies showing reduction in hemodynamic AEs, whereas other studies have found no difference in serious AEs with the use of ketamine compared to etomidate.^27^,^28^ Furthermore, others have found a higher incidence of peri-intubation hypotension when ketamine was received compared to etomidate.^29^ Based on the current data available, ketamine and etomidate are both reasonable induction agents for hemodynamically compromised patients.

It is well understood that the anatomically difficult airway places patients at risk for AEs.^1^,^3^ Our findings suggest that the physiologically difficult airway is at least as important as the anatomically difficult airway. Furthermore, having both an anatomically and physiologically difficult airway places the patient at an additive risk for AEs and decreased FPS-AE. Physicians should approach the physiologically difficult airway with the same level of concern and preparation to mitigate risks as the anatomically difficult airway. Research is urgently needed to determine the best approach to attenuate these risks.

## LIMITATIONS

This study has several important limitations that must be considered. This was a single-center study in an academic medical center where residents performed the vast majority of intubations, limiting the ability to generalize the findings to other clinical settings. Even though 87% of forms were collected in real time, the data are subject to self-report, recall bias, and under-reporting of AEs. However, if AEs were under-reported, it is likely that they would be equally under-reported in both the anatomically and physiologically difficult airway groups. Additionally, there were potential important confounders such as level of training and indication for intubation that we attempted to account for by adjusting in the regression model. However, there could be important unknown confounders that we cannot account for, such as preintubation vital signs, vasopressor use, and fluid resuscitation.

We also did not document what modifications were made when an anatomically or physiologically difficult airway was predicted. However, in this training program, residents are instructed to perform an anatomic and physiologic difficult airway assessment prior to intubation to develop a strategy to address these issues. Device selection, particularly the use of VL, could be perceived as advantageous for the airway operator considering they were not blinded to the presence of an anatomically or physiologically difficult airway. This selection may have contributed to increased FPS-AE. However, our ED primarily uses VL as a first-line device so any advantage was likely present among all of the groups.

An additional limitation is that an independent reviewer did not determine the presence of difficult airway characteristics, potentially leading to bias that was not considered. Difficult airway assessment is subjective and this would not be feasible in our clinical environment. Furthermore, data collection forms were completed by the operator following the procedure and thus the procedure itself may have impacted what was documented on the form. Ideally, the operator would complete the airway form prior to intubation. However, given time constraints this is not feasible in our ED.

Since patients with physiologically difficult airway characteristics had similar characteristics to AEs, namely hypoxemia and hypotension, it was critical these definitions were clarified prior to the intubation attempt. Airway operators were instructed that if they had a physiologically difficult airway, hypotension or hypoxemia were not considered AEs unless there was improvement with subsequent reduction of blood pressure or oxygen saturation after the intubation event. All patients undergoing RSI in the ED were included in the study. Patients who had return of spontaneous circulation that required RSI to facilitate intubation had their anatomy and physiology assessed by the operator at the time of intubation and thus this was independent of their prehospital course.

## CONCLUSION

In this analysis of continuous quality improvement data from an academic ED, first pass success without adverse events decreased similarly for patients with either an anatomically or physiologically difficult airway. Optimization strategies to improve first pass success without adverse events for patients with physiologically difficult airways should be studied in randomized controlled trials.

## Figures and Tables

**Table 1:** Difficult airway characteristics.

Anatomically difficult airway characteristics	Physiologically difficult airway characteristics
Cervical immobility	Hypoxemia
Facial/neck trauma	Hypotension
Airway edema	Metabolic acidosis
Small mandible	Right ventricular failure
Obesity	
Large tongue	
Short neck	
Restricted mouth opening	
Blood in the airway	
Vomit in the airway	

**Table 2:** Patient, intubation, and operator characteristics.

Groups (number of observations)	None (210)	ADA (649)	PDA (186)	Both (468)
Age, median (IQR), y	51 (33 – 62)	49 (32 – 62)	59 (40 – 71)	54 (38 – 66)
Gender				
Male, no. (%)	135 (64)	441 (68)	121 (65)	313 (67)
Medical/trauma, no. (%)				
Medical	189 (90)	328 (51)	181 (97)	300 (64)
Trauma	21 (10)	321 (49)	5 (3)	168 (36)
Reason for Intubation, no. (%)				
Airway protection	156 (74)	519 (80)	74 (40)	282 (60)
Cardiac arrest*	3 (1)	5 (0.8)	5 (3)	18 (4)
Patient control	24 (12)	65 (10)	6 (3)	14 (3)
Shock	0 (0)	1 (0.2)	8 (4)	17 (4)
Respiratory failure	27 (13)	59 (9)	93 (50)	134 (29)
Specific ADA, no. (%)				
Cervical immobility	N/A	304 (47)	N/A	
Facial/neck trauma		125 (19)		
Airway edema		26 (4)		
Small mandible		61 (9)		
Obesity		294 (45)		
Large tongue		100 (15)		
Short neck		84 (13)		
Restricted mouth opening		56 (9)		
Blood in airway		129 (20)		
Vomit in airway		90 (14)		
Specific PDA characteristics, no. (%)				
Hypoxemia	N/A	N/A	105 (56)	259 (55)
Hypotension			90 (48)	251 (54)
Metabolic acidosis			59 (32)	136 (29)
RV failure			5 (3)	6 (1)
NMBA used, no. (%)				
Succinylcholine	118 (56)	451 (69)	71 (38)	267 (57)
Rocuronium	92 (44)	198 (31)	115 (62)	201 (43)
Induction agent used, no. (%)				
Etomidate	173 (82)	603 (93)	145 (78)	412 (88)
Ketamine	16 (8)	35 (5.3)	38 (20.5)	52 (11)
Midazolam	5 (2.5)	3 (0.5)	1 (0.5)	3 (0.6)
Propofol	15 (7)	8 (1.2)	2 (1)	1 (0.2)
Other	1 (0.5)	0 (0)	0 (0)	0 (0)
Operator specialty, no. (%)				
EM	181 (86)	579 (89)	171 (92)	412 (88)
EM-PEDS	29 (14)	70 (11)	15 (8)	56 (12)
Operator PGY EM, no. (%)				
PGY-1	43 (20)	96 (15)	40 (21)	63 (13)
PGY-2	82 (39)	269 (41)	76 (41)	180 (39)
PGY-≥ 3	85 (41)	284 (44)	70 (38)	225 (48)
Device Used, no. (%)				
DL	23 (11)	39 (6)	14 (8)	15 (3)
SG VL	132 (63)	322 (50)	149 (80)	275 (59)
HA VL	55 (26)	288 (44)	23 (12)	178 (38)

* = cardiac arrest patients who had return of spontaneous circulation.

*None*, no difficult airway characteristic; *ADA*, anatomically difficult airway; *PDA*, physiologically difficult airway; *Both*, both anatomically and physiologically difficult airway characteristic; *IQR*, interquartile range; *NMBA*, neuromuscular blocking agent; *EM*, emergency medicine residents; *EM-PEDS*, combined emergency medicine and pediatric residents; *PGY*, postgraduate year; *DL*, direct laryngoscopy; *SG VL*, standard geometry video laryngoscope; *HA VL*, hyperangulated video laryngoscope.

**Table 3:** First-pass success without adverse events in all cohorts.

Groups (n)	FPS-AE % (n)	95% CI	% Difference (95% CI)
None (210)	92.4 (194/210)	87.9% to 95.6%	[Reference]
ADA (649)	82.1 (533/649)	78.9% to 85.0%	−10.3% (−14.8% to −5.6%)
PDA (186)	81.7 (152/186)	75.4% to 87.0%	−10.7% (−17.3% to −4.0%)
Both (468)	70.9 (332/468)	66.6% to 75.0%	−21.4% (−27.0% to −16.0%)

*FPS-AE*, first-pass success without adverse events; *CI*, confidence interval; *None*, no difficult airway characteristic; *ADA*, anatomically difficult airway; *PDA*, physiologically difficult airway; *Both*, both anatomically and physiologically difficult airway characteristics.

**Table 4:** Multivariable regression analysis of predictors of first-pass success without adverse events.

FPS-AE	Odds ratio	95% CI	*p-*value
None	[Reference]		
ADA	0.37	0.21 – 0.66	0.001
PDA	0.36	0.19 – 0.67	0.001
Both	0.19	0.11 – 0.33	<0.001
Training year			
PGY1	[Reference]		
PGY 2	1.27	0.87 – 1.84	0.214
PGY ≥ 3	1.40	0.96 – 2.03	0.078
Trauma	0.90	0.67 – 1.22	0.511
Age (years)	1.00	0.99 – 1.01	0.949
Device			
DL	[Reference]		
SG VL	1.77	1.04 – 3.03	0.036
HA VL	1.74	1.00 – 3.02	0.049

*FPS-AE*, first-pass success without adverse events; *CI*, confidence interval; *None*, no difficult airway characteristic; *ADA*, anatomically difficult airway; *PDA*, physiologically difficult airway; *Both*, both anatomically and physiologically difficult airway characteristics; *PGY*, postgraduate year; *DL*, direct laryngoscopy; *SG VL*, standard geometry video laryngoscope; *HA VL*, hyperangulated video laryngoscope.

**Table 5:** Adjusted odds ratios for individual difficult airway predictors.

FPS-AE	Odds ratio	95% CI	*p-*value
Training Year			
PGY1	[Reference]		
PGY2	1.27	0.86 – 1.87	0.228
PGY ≥ 3	1.45	0.98 – 2.14	0.062
Trauma	0.86	0.54 – 1.38	0.536
Age	1.00	0.99 – 1.01	0.719
Device			
DL	[Reference]		
SG VL	1.91	1.09 – 3.13	0.023
HA VL	1.77	1.00 – 3.12	0.050
DAC			
None	[Reference]		
Blood in the airway	0.52	0.35 – 0.78	0.001
Vomit in the airway	0.84	0.55 – 1.29	0.436
Short neck	1.23	0.79 – 1.92	0.349
Cervical immobility	1.05	0.68 – 1.63	0.824
Small mandible	0.56	0.35 – 0.89	0.014
Obesity	0.82	0.59 – 1.12	0.238
Airway edema	0.70	0.35 – 1.39	0.308
Facial/neck trauma	0.94	0.58 – 1.52	0.812
Large tongue	0.44	0.29 – 0.65	<0.001
Restricted mouth opening	0.32	0.21 – 0.50	<0.001
Hypoxemia	0.35	0.26 – 0.48	<0.001
Hypotension	1.26	0.90 – 1.78	0.173
Metabolic acidosis	0.96	0.63 – 1.44	0.834
RV failure	0.36	0.10 – 1.35	0.130

*FPS-AE*, first-pass success without adverse events; *CI*, confidence interval; *PGY*, postgraduate year; *DL*, direct laryngoscopy; *SG VL*, standard geometry video laryngoscope; *HA VL*, hyperangulated video laryngoscope; *DAC*, difficult airway characteristic; *RV*, right ventricular.
